# From Serendipity to Strategy: Rationalizing Molecular Glue Discovery and Proximity-Induced Pharmacology through Chemical Biology

**DOI:** 10.1021/jacs.5c12299

**Published:** 2026-02-02

**Authors:** Janine L. Gray, Zhangping Xiao, Vanessa V. Rogga, Xinyue Zhang, Edward W. Tate

**Affiliations:** † Department of Chemistry, Molecular Sciences Research Hub, 4615 Imperial College London, London W12 0BZ, U.K.; ‡ Laboratory for Synthetic Chemistry and Chemical Biology Limited Units, 15/F., Building 17W, Science Park, Hong Kong SAR, China; § The Francis Crick Institute, London NW1 1AT, U.K.

## Abstract

Molecular glues represent a new paradigm in drug discovery, stabilizing novel protein–protein interactions between two proteins to elicit targeted cellular outcomes. Historically discovered through serendipity, molecular glue identification is becoming increasingly systematic. In this perspective, we discuss the current advances and challenges of this field, highlighting the transition toward rational discovery through the convergence of four complementary approaches. Innovations in library design and screening platforms are expanding access to glue-relevant chemical space, guided by a deeper mechanistic understanding of proximity-induced pharmacology. These efforts are further enabled by functional genomic approaches that reveal gluable interfaces. Finally, the integration of chemical and biological data through machine learning is beginning to support rational de novo glue design.

## Graphical abstract

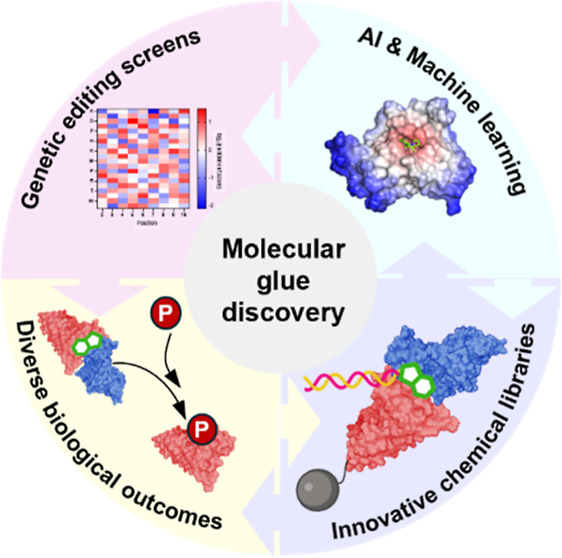

## 1 Introduction

Molecular glues (MGs) represent a powerful approach to broaden the scope of small-molecule therapeutics by stabilizing protein–protein interactions (PPIs), often enabling selective degradation or functional modulation of targets previously considered intractable.^1^ They achieve this by remodeling a key region of the interface to create a neomorphic surface, enforcing structural complementarity where none existed or was only weakly favored. This ability to harness an interaction surface through a drug-like molecule opens new avenues to engage proteins that lack a conventionally ligandable site and unlocks diverse forms of proximity-induced pharmacology (PIP) while avoiding the developability challenges associated with large PPI inhibitors or peptides. In the 35 years since the discovery of the first natural product molecular glues—including rapamycin, whose mechanism was elucidated by Schreiber and colleagues,^2^ as well as auxin^3^ and the fusicoccanes^4^—the scope of MGs has evolved to include proximity-inducing small molecules and blockbuster drugs such as pomalidomide, catalyzing parallel advances in bifunctional modalities such as Proteolysis Targeting Chimeras (PROTACs).

However, despite early promise and notable successes in specific cases, new classes of MGs remain difficult to discover. Unlike bifunctional molecules, which can be rationally assembled from known ligands and modular linkers, MGs must induce productive ternary complexes without prior evidence for binding affinities or pre-existing interactions among the three components (Figure 1A). A key feature of this process is cooperativity, where binding of the MG enhances the interaction between the effector and protein of interest (POI), selectively stabilizing the ternary complex relative to each binary interaction. This stands in contrast to bifunctional molecules, which can still retain activity with non- or anticooperative binding due to their bivalent nature. For glues, the typically high dissociation constants of binary interactions imply that their binding mechanism is cooperative. However, cooperative effects are typically context-dependent and thus challenging to predict. As a result, MG discovery remains a highly complex problem, requiring simultaneous navigation of chemical space, protein interface geometry, and biological function.

**Figure 1:**
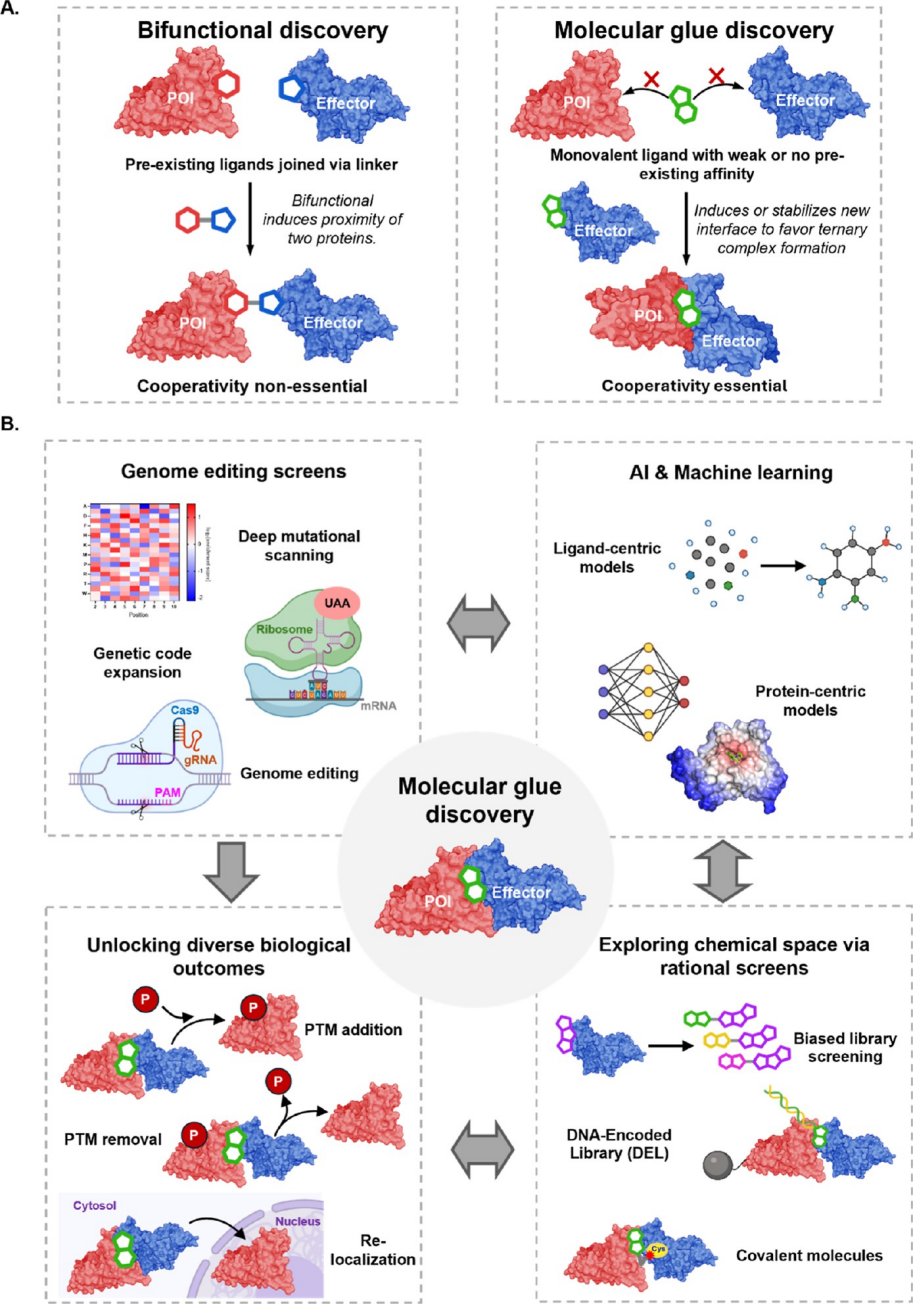
Molecular glue discovery. (A) Bifunctional molecules consist of two pre-existing ligands for the effector and POI, joined by a linker chain and can function catalytically without requiring cooperativity. Molecular glues are monovalent small molecules with weak or no pre-existing binary binding. They cooperatively induce or stabilize a new protein–protein interface, forming a ternary complex with complementary neomorphic surfaces between the effector and POI. (B) Key developing areas—rational chemical screens, new proximity-induced pharmacology mechanisms, genetic screening and AI/ML—are accelerating MG discovery.

Recent progress in MG discovery draws on advances across four complementary domains (Figure 1B). First, innovations in chemical library design, including biased and encoded formats, covalent probes, and direct-to-biology (D2B) platforms, are enabling focused and efficient exploration of glue-relevant chemical space. Second, these strategies are increasingly guided by a deeper understanding of PIP, which shows how neomorphic interactions can drive not only degradation but also reprogram localization, signaling, and post-translational modifications (PTMs). Third, complementary advances in functional genomics and protein engineering, though mainly demonstrated in bifunctional degrader systems, offer the potential to discover novel PIP modalities, cryptic interaction surfaces and previously hidden gluable sites, providing biological context that directly informs screening and validation. Finally, integration of chemical and biological data through molecular modeling and machine learning is beginning to uncover predictive features and emerging design principles, supporting prioritization of protein pairs, modeling of cooperativity, and ultimately, the rational design of MGs from first principles.

MG discovery defines a chemical biology paradigm in which insights from cell biology and medicinal chemistry are fundamentally interdependent, and together these domains generate the data and mechanistic understanding to support predictive computational models. Systematic reviews summarizing the progress in MG discovery and the broader PIP field have been presented in other review articles.^1,5–9^ Here, we discuss how the integration of these four areas has the potential to transform a historically largely empirical pursuit into a systematic and programmable approach to therapeutic discovery.

## 2 Adapting Chemical Space for Molecular Glue Discovery

The discovery of molecular glues has traditionally been driven by serendipity. In theory, an ideal screening platform would identify ternary complexes between a POI, effector protein, and small molecule in high-throughput. In practice, the simultaneous resolution of novel PPIs and systematic exploration of glue-relevant chemical space presents a formidable challenge. The dual complexity of accessing diverse and relevant chemical space, itself a major barrier in traditional ligand discovery for difficult targets, combined with the equally challenging “biochemical space” of mutually compatible PPIs, greatly exceeds the capabilities of conventional screening approaches.

Emerging chemistry-centered strategies have the potential to address the inherent complexity of glue discovery and offer feasible alternatives to traditional high-throughput screening (HTS) approaches. Here we consider three emerging approaches which trade a degree of flexibility against a demonstrated capacity for MG discovery: focusing chemical exploration on known glue-like space through biased libraries; expanding chemical space while simultaneously selecting for cooperativity using encoded libraries; and incorporating covalent warheads to enforce specific proximity-driven interactions.

### 2.1 Biased Library Screening Facilitates Glue Discovery

Libraries featuring scaffolds biased toward a specific protein family—such as kinases or GPCRs—are widely used in drug discovery to narrow chemical space and significantly increase hit rate in HTS.^10^ As a step toward rational molecular glue discovery, biased libraries aim to improve hit rates for a specific effector or POI by strategically incorporating additional motifs into a known ligand scaffold. These libraries are typically designed and synthesized de novo, focusing on solvent-exposed vectors to enable ternary complex formation without compromising the original binding affinity. By narrowing chemical space and anchoring one interaction partner, biased libraries help address the “three-body problem” of glue discovery, allowing functional screening in cells against a defined POI or effector. This approach simplifies the deconvolution of mechanisms, as one binding partner is already known. Moreover, dense sampling of target-focused chemical space provides immediate structure–activity relationship (SAR) data in the primary screen, supporting identification of trends that increase confidence in the molecular mechanism of action (MoA).

#### 2.1.1 Effector-Biased Libraries

The biased chemical library approach has been exemplified in cellular screens using effector-centric libraries (Figure 2A). For example, biased immunomodulatory drug (IMiD) libraries applied to screens for changes in cellular morphology led to the discovery of an MG with cereblon (CRBN)-dependent neosubstrate degradation (FL2–14, Table 1).^11^ Biasing the assay itself toward MG discovery also has potential; for example, screening specific mechanisms of protein degradation linked to cell viability in panels of engineered cell lines has led to MG discovery even for relatively unbiased chemical libraries.^12^ In one recent proof-of-concept taking advantage of the tendency of E3 ligase components to undergo autodegradation in the absence of a substrate, “ligase-tracing” screens identified ternary complex formation indirectly in cells through stabilization of a Nanoluciferase-tagged E3 ligase.^13^ Aryl-sulfonamide dRRM-1 (Table 1) was discovered by DCAF15 stabilization through recruitment of neosubstrate RBM39. However, mechanistic deconvolution remains challenging; cellular screens can be difficult to scale, particularly those depending on proteomic analysis to discover targets of degradation and are constrained by endogenous expression in the chosen cell model. As such, this approach may be most effective when combined with biased chemical libraries.^14^

**Figure 2:**
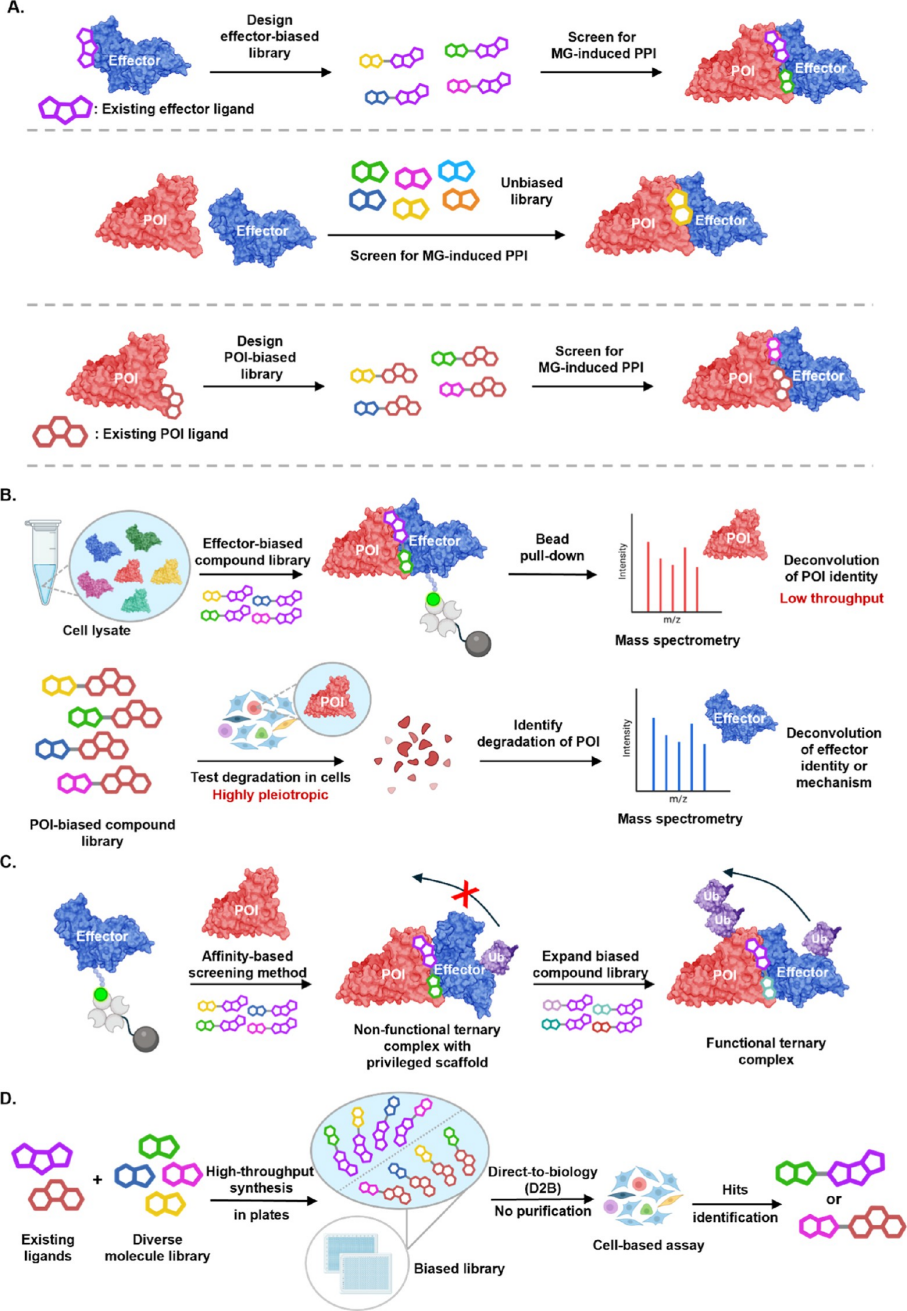
Biased screening strategies. (A) Comparison of unbiased HTS libraries with biased libraries. Effector-centric libraries enable screening against a wide range of POIs, while POI-centric libraries increase the likelihood of identifying novel effectors that induce a desired cellular effect. (B) Two contrasting screening strategies are shown: one identifies novel POIs for a specific effector (limited by low-throughput proteomics or affinity-MS), while the other starts with POI degradation and requires challenging deconvolution of the underlying mechanism. (C) Affinity-based methods may result in the identification of nonfunctional ternary complexes. Screening an expanded biased library against the complex may identify neomorphic interfaces that result in desired functional outcome. (D) D2B technology offers a rapid and automated platform for MG development, enhancing screening efficiency.

**Table 1:**
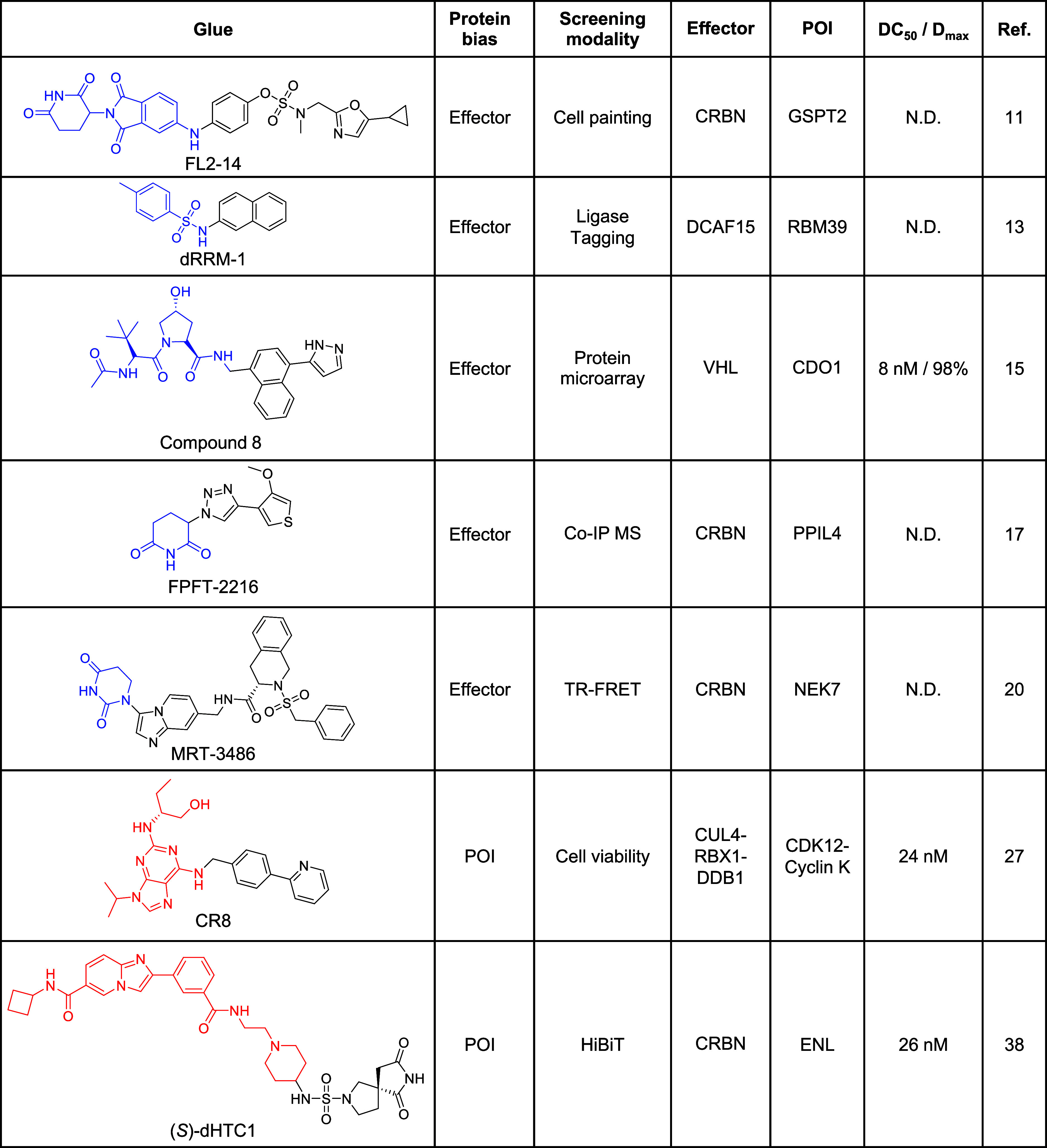
Molecular Glues Discovered in Biased Library Screens^a^

a Parts of the scaffold that originate from a known glue or ligand are highlighted in blue (effector ligand) or red (POI ligand). N.D. = not determined.

In affinity-based MG discovery, a tagged effector protein is used as bait to isolate interacting partners from cell lysates or recombinant protein libraries (Figure 2B). These approaches select for ternary complex formation and can reveal low-abundance or isoform-specific POIs that might be overlooked in phenotypic screens. For example, a protein microarray using a small VHL-biased ligand set identified CDO1, a protein with generally low expression levels, as a neosubstrate in a glue–VHL ternary complex (compound 8, Table 1).^15^ While the agnostic nature of effector-based libraries may enhance the discovery of neosubstrates, it can lead to the identification of POIs with no immediate therapeutic relevance, as exemplified by CDO1. Nonetheless, immunoprecipitation MS (IP-MS)^16,17^ studies with IMiD analog libraries identified new CRBN substrates including novel zinc fingers and POIs lacking canonical degron motifs (e.g., FPFT-2216 as a MG for PPIL4, Table 1), while enabling rational selection of POIs which natively interact with a specific effector for subsequent focused screens (see Section 2.2).

The small number of E3 ligases with known ligands remains a key limitation for effector-biased libraries, with most MG and PROTAC strategies still centered on VHL and CRBN due to their strong track record in targeted protein degradation (TPD) and consequent investment in chemistry.^18,19^ Discovery of new E3 ligase ligands which can support functional E3 ligase activity remains challenging since many substrate binding sites are either not known or are not readily liganded by simple small molecules, and ligands at other sites may not induce degradation. Indeed, conversion of gluing to degradation remains a challenge for IMiD MGs where co-IP MS frequently identifies POIs that are not degraded by the MG in the cell,^17^ suggesting stabilization of a ubiquitination-inactive ternary complex.

However, a recent publication illustrates that nonfunctional ternary complexes, initially identified through affinity-based screens, can be converted into ubiquitination–active complexes by screening against an extended biased CRBN-focused library, increasing access to differential PPI interfaces (Figure 2C, MRT-2386 in Table 1).^20^ While this highlights the potential for affinity-based approaches, the success relied on a large proprietary library for CRBN which may not be generalizable to other effectors or induced proximity mechanisms. Although this is a limitation of the effector affinity approach, the identification of MG activity remains important for future design strategies, including guiding machine learning approaches (Section 5). In parallel, emerging systematic methods for identification of degradation-competent ligandable effector sites would enable a wide range of opportunities (Section 4).^21^

Beyond classical molecular glue degraders, other privileged scaffolds such as fusiccocane 14–3–3 glues highlight the potential of effector-biased library design.^4^ Unlike typical MGs, 14–3–3 glues do not induce neomorphic PPIs or engage an “effector” as described previously. Instead, fusicoccanes bind at the interface of the phosphorylation reader 14–3–3 and its client proteins, modulating pre-existing PPIs. Many enzymes are functionally regulated via complex formation with 14–3–3.^22^ Alternatively, 14–3–3-POI formation can impact POI localization or stabilization.^4,23^ The fusicoccane privileged scaffold has been amenable to SAR studies, expanding client protein recruitment in a manner analogous to biased CRBN libraries.^24^ Recent 14–3–3 glues have been discovered or optimized through structure-based design^24,25^ and in silico^26^ approaches, underscoring the value of computational tools in guiding library development (Section 5). While the lack of neosubstrate recruitment has prompted ongoing debate as to whether they meet MG definitions or simply shift pre-existing binding equilibria, they exemplify the broader potential to modulate diverse cellular processes using glue-like small molecules.

#### 2.1.2 Protein of Interest (POI)-Biased Libraries

POI-biased ligand libraries offer a promising alternative to effector-biased libraries (Figure 2A). In the context of TPD, these libraries identify compounds that cause POI degradation in cellular screens without a predefined effector, with the potential to discover effectors in diverse degradation pathways (Section 3). While this area is currently under-explored, there are clear opportunities to repurpose compounds previously deprioritized in traditional inhibitor discovery campaigns due to lower binding affinity (*K*_D_) or potency (IC_50_), but may nonetheless possess cryptic MG activity. Following the serendipitous discovery of kinase inhibitor molecular glue degraders of Cyclin K via formation of DDB1-CDK12-CyclinK complex, such as CR8 (Table 1),^27,28^ systematic cellular screens of established kinase inhibitors have indeed uncovered rare examples of degraders.^29^ However, in many cases, the mechanism of degradation remains unclear and is often difficult to tease apart from the pharmacological impacts of kinase inhibition (Figure 2B). The diverse ways in which the level of a POI could be reduced in cellular screens—notably, toxicity leading to reduced synthesis rates—results in a high false positive rate. While careful controls can mitigate some of these issues,^30^ the true positive rate generally remains low.

#### 2.1.3 Direct-to-Biology (D2B) Platforms Enable Biased Molecular Glue Discovery

Large-scale biased library generation on-demand requires miniaturized, robust and automated chemistry, which is typically limited by the need for rounds of purification which are difficult to scale. D2B workflows, in which crude reaction mixtures are screened directly in cells without purification, are gaining popularity for efficient generation and screening of biased libraries at scale (Figure 2C). Although limited to transformations offering sufficient conversion and commercial building block diversity (e.g., amide formation, cross couplings, click chemistries), cellular assays can be remarkably robust to interference from unpurified reaction mixtures. Potential artifacts such as byproducts, starting materials, or off-target toxicity can typically be controlled for separately.^31^ Furthermore, low conversion does not necessarily compromise MG discovery due to their cooperativity and potential catalytic degradation activity.

D2B strategies are well-exemplified for PROTAC discovery, where modular assembly of ligands into bifunctional molecules aligns with combinatorial synthesis.^32–34^ In contrast, biased MG libraries require more bespoke approaches, and published examples to date have been limited to IMiD scaffolds recruiting CRBN which have proven remarkably tolerant to modification.^35–37^ Extending D2B to other glue scaffolds may be more challenging, as incorporation of larger groups or linkers may generate bifunctionals lacking cooperativity, or disrupt binding to the original protein. A recent example currently in preprint led to discovery of (*S*)-dHTC1 (Table 1) with properties intermediate between a MG and a PROTAC, while showing strong cooperative binding between the POI (ENL) and CRBN.^38^

### 2.2 Encoded Library Screening Enables Selection for Cooperativity

Screening technologies that explore chemical space more efficiently than traditional HTS have the potential to reduce dependence on pre-existing ligands for MG discovery. Encoded libraries, such as DNA-encoded libraries (DELs) or mRNA display, consist of large pools of small molecules or peptides, each uniquely tagged with a nucleic acid barcode. These barcodes serve as molecular identifiers during selection and recovery, allowing rapid and high-throughput screening of billions of compounds against defined targets (Figure 3A).^39,40^ DNA and peptide encoded libraries have been successfully applied to bifunctional ligand^41^ and MG discovery,^42–45^ enabling target-based screening for compounds promoting ternary complex formation between a specific effector and POI in a single assay.

**Figure 3:**
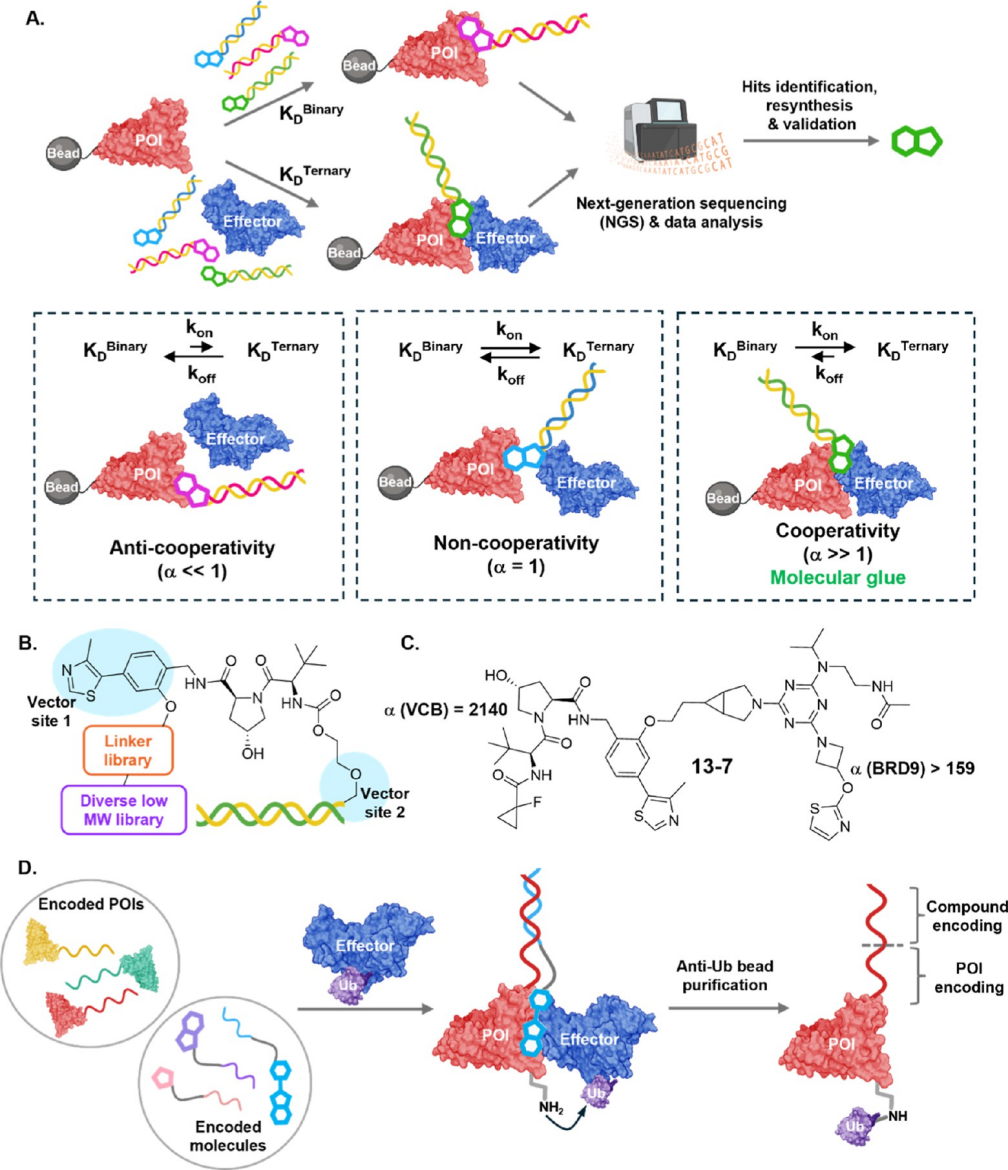
Encoded libraries for MG discovery and cooperativity assessment. (A) Encoded libraries consist of pools of candidate molecular glues, each tagged with a unique nucleic acid barcode. Affinity-based enrichment using magnetic bead-bound POI, in the presence and absence of an effector protein, enables identification of compounds that preferentially bind the ternary complex. The degree of preferential binding can be quantified as a cooperativity factor (α), allowing discrimination between true molecular glues and traditional bifunctional ligands. (B) Example of a VHL-biased chemical inducers of proximity DNA encoded library (CIP-DEL) scaffold design. (C) Compound 13–7, an MG identified through the CIP-DEL approach, exhibits enhanced binding affinity in ternary complex compared to the binary complex. (D) Dual-encoded libraries for POI and small molecules enable the development of functional DEL screens. POIs are identified following small molecule-induced ternary complex formation and ubiquitination.

A key innovation for MG discovery has been the design of screens that select for cooperativity. For MGs, cooperativity refers to the increase in binding affinity resulting from the formation of a ternary complex relative to the individual binary protein interactions. High cooperativity, reflected in strong affinity for the ternary complex, is often essential for cellular activity of molecular glues. However, while binary MG-protein interactions are often weak, undetectable or even absent when assessed individually, they can nonetheless be essential for remodeling protein surface enabling ternary complex assembly. In contrast, cooperativity for bifunctional molecules, such as PROTACs, is more nuanced, in part due to differing definitions. For PROTACs, the term typically describes how binding of one end of the molecule influences binding of the other, rather than the overall stability of the ternary complex.^5^ Noncooperative and anticooperative/antagonistic PROTACs may still induce degradation because the low-affinity ternary complex can permit ubiquitination if catalysis is highly efficient.^46–48^ Alternatively, cooperative or synergistic PROTACs may not result in POI degradation if they prevent catalytic cycles.^49^

MG cooperativity aligns naturally with affinity-based enrichment screens used for encoded libraries, since cooperative binding should enhance recovery of low-abundance ternary complexes at the expense of binary complexes. By enriching compounds on POI-immobilized beads in the presence and absence of the “presenter” protein (recruited binder—in this case, the effector), ratios for ternary to binary complex enrichment can be determined. These enrichment scores may correlate with cooperativity, enabling the selection of compounds which exhibit high cooperativity (α ≫ 1). For example, a biased DEL library incorporating known VHL interaction motifs (Figure 3B) identified hits with high cooperative binding for VHL and BRD9 (Figure 3C).^43^ Since the ternary complex is of much higher affinity than either binary complex, these compounds did not exhibit a “hook” effect, a phenomenon commonly observed with bifunctional molecules such as those discovered in a related DEL screen for VHL-BRD4.^41^ A similar strategy using a 10^8^ member phage-display library identified cooperative α-helical peptides, and these methods could likely be adapted for other encoded technologies such as mRNA display.^39,44^

In the target-based screens described above, the protein scope and chemical space are typically restricted to increase the likelihood of identifying cooperative binders. However, this requires preselection of both the effector and POI, and the success of the screen depends on the inherent compatibility of the chosen pair, a choice often influenced by known or latent affinity. This presents a challenge in rationally selecting effector-POI pairs, particularly since biochemical cooperativity may not always correlate with the desired cellular activity. However, a recent cell-based screen of a VHL-DEL library demonstrated that glue-dependent degradation correlated more strongly with ternary complex dissociation rate (*k*_off_) than association rate (*k*_on_).^50^ Slow complex dissociation led to increased target degradation, while rapid dissociation could not be overcome by prolonged incubation or higher compound concentrations. This suggests that screening approaches favoring cooperative binding (e.g., DELs or co-IP MS) may enrich for compounds with slow *k*_off_ rates, which could increase the likelihood of identifying functional glue degraders.

Cooperative glue discovery has not been exemplified to date using unbiased encoded libraries. The DELs described above are biased toward VHL, for which the ligand bears two exit vectors, one enabling chemical derivatization and the other accommodating DNA barcoding (Figure 3B). While this scaffold is well-suited to encoded screening formats, the resulting VHL-based MGs are relatively large, raising concerns about pharmacokinetic properties more typical of bifunctional molecules. Other E3 ligase ligands, such as thalidomide, are smaller but may not tolerate such dual modifications. Moreover, the buried nature of many glue-binding sites at the interface complicates the incorporation of linkers or nucleic acid barcodes without disrupting binding. A potentially more promising strategy is the incorporation of covalent handles into DELs, enabling irreversible or prolonged engagement through reaction with accessible nucleophilic residues.^51^ This can increase residence time and reduce reliance on optimal vector positioning, thereby facilitating the design of unbiased glue libraries, potentially informed by AI/ML tools to guide scaffold selection and covalent warhead placement.^52^

The discovery of cooperative peptides may be more tractable due to their larger interaction surfaces and capacity to span effector-POI interfaces. Peptides can be identified via encoded technologies or designed using AI/ML approaches based on sequence. For example, “Trimerizer” peptides have identified ligandable E3 ligases across all four major ligase families.^44^ Using mRNA display, a macrocyclic peptide glue was discovered that stabilizes an inactive homodimeric form of MCL1, a key antiapoptotic protein implicated in cancer.^45^ Although peptides often suffer from poor pharmacokinetic properties, they may serve as valuable probes in displacement assays to identify cooperative small-molecule binders targeting the same interface, and they can be expressed and screened directly in cells.^53^ The use of nucleic acid barcodes restricts encoded screens to effector-POI pairs that do not natively bind DNA or RNA, limiting some applications; emerging solutions to this challenge avoid tag interference with so-called “solid state” DELs displayed on beads, with a greatly reduced ratio of nucleic acid tag to ligand.^54^ Other emerging approaches for MG discovery adapt DELs for multiplexed functional screens such as in vitro ubiquitin transfer assays to directly assess POI ubiquitination and improve functional hit validation (Figure 3D),^55^ or for in-cell target-based screens through microinjection of the library directly into large oocytes engineered to translate a desired POI.^56^

### 2.3 Proximity-Driven Covalent Glues

#### 2.3.1 Effector-Reactive Covalent Glues

The incorporation of covalency offers a powerful strategy to increase the tractability of the proteome for MG discovery. Most examples involve covalent modification of the effector by the glue. For instance, existing E3 ligase ligands have been adapted to include covalent warheads, allowing modification of the E3 independently of POI binding (Figure 4A). Covalent ligands for CRBN^57^ and VHL^58^ have been successfully used in PROTACs (Table 2), and novel covalent E3 ligands with glue activity have been identified through covalent fragment screening.^59^ However, the frequent use of unoptimized warheads and scaffolds in the latter approaches brings a severe risk of off-target protein engagement, pleiotropic mechanisms, and cellular toxicity.

**Figure 4:**
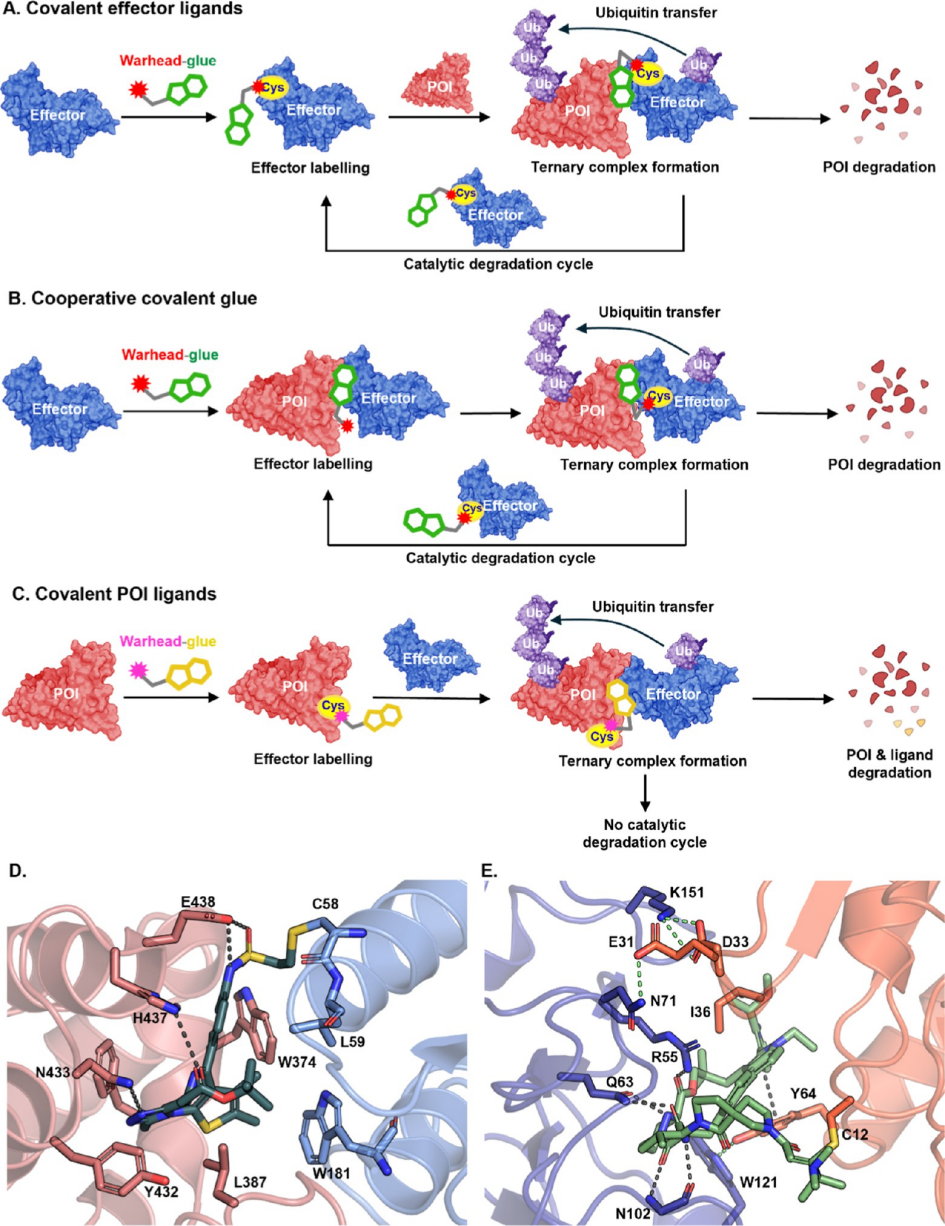
Covalent glue mechanisms. (A) Noncooperative covalent glues: effector ligands, which may also be MGs, bearing a reactive warhead forms covalent bond with the E3 ligase independently of POI-induced structural changes; low stoichiometry effector modification may catalyze multiple degradation cycles while leaving most of the ligase unmodified to preserve native function. (B) Cooperative covalent glues: covalent bond formation occurs only upon cooperative ternary complex assembly; POI binding is required to enable selective covalent modification of the effector, and thus the MG is active only in the presence of the POI. (C) Covalent POI ligands: these compounds covalently modify the POI and can induce effector recruitment but lack the catalytic degradation mechanism associated with covalent effector glues. (D) Example of effector-targeting cooperative covalent glue (PDB: 8G46). MMH2 (dark green sticks, Table 2) is covalently bound to C58 of DCAF16 (blue). Key hydrogen bonds to BRD4 (pink) situated within a large hydrophobic binding pocket are depicted by dashed gray lines. The ligand forms minimal interactions with DCAF16 beyond the covalent bond. (E) Example of POI-targeting covalent glue (PDB: 8G9P). RMC-4998 (light green sticks) is covalently bound to C12 of KRas^G12C^. Key interactions with residues from KRas^G12C^ (orange) and cyclophilin A (dark blue) are depicted by dashed gray lines. Induced interactions within the neomorphic interface generated by ternary complex formation are shown as dashed lime-green lines.

**Table 2:**
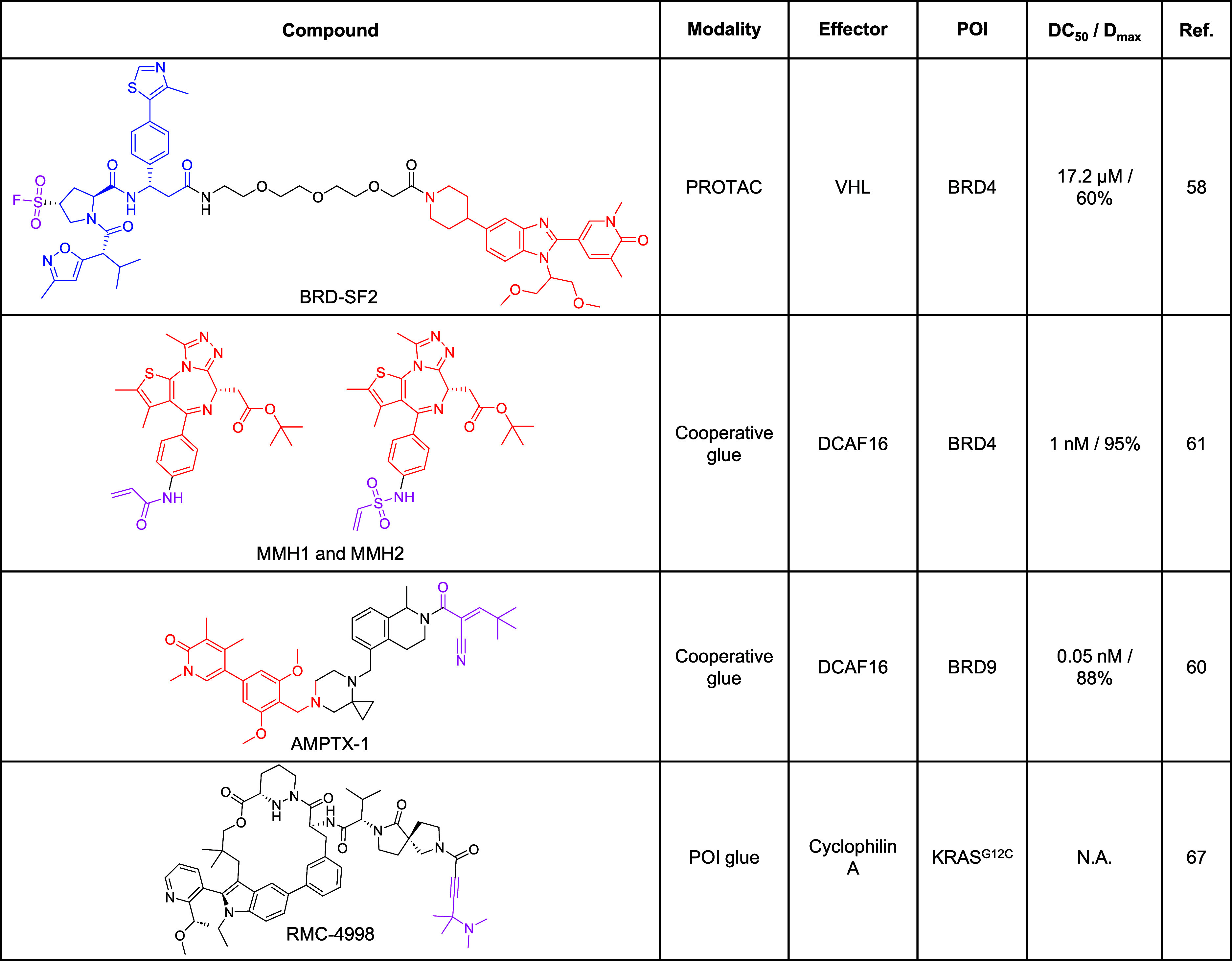
Representative Examples of Covalent Degraders and MGs^a^

a Parts of the scaffold that originate from a known glue or ligand are highlighted in blue (effector ligand) or red (POI ligand). The covalent warhead is highlighted in pink. N.A.—not applicable.

In contrast, cooperative covalent MGs combine synergistic reversible binding with proximity-induced covalency (Figure 4B). These glues initially form a reversible ternary complex with the effector and POI, and the neomorphic surface induced upon complex formation aligns the covalent warhead for selective reaction with a nucleophilic residue on the effector. Cooperative covalent glues have been found using libraries based on BRD9 and BRD4 ligands, (AMPTX-1, MMH1 and MMH2, Table 2) whereby incorporation of a covalent modality in the POI ligand results in covalent modification of DCAF16 following ternary complex formation.^60,61^ Unlike conventional covalent E3 ligands, which require high affinity for the effector, these glues can exhibit low intrinsic affinity for the ligase. For example, DCAF16-targeting cooperative glues weakly bind to the E3 on their own and only achieve covalent modification in the presence of the POI (Figure 4D). This mechanism resembles the protein-templated labeling seen in activity-based probes (ABPs), in which a covalent warhead targeting an active site residue is appended to a high-affinity ligand;^62^ while ABPs only label their target when it is catalytically active, cooperative covalent MGs only modify the effector in the POI-effector ternary complex, mitigating the risk of promiscuous modification.

This discovery paradigm is well-aligned with affinity-based encoded library screening strategies (Sections 2.1 and 2.2); the prolonged residence time of cooperative covalent glues may enhance recovery in DEL screens, and D2B platforms enable rapid synthesis of POI ligand libraries bearing diverse warheads.^63^ This approach allows systematic tuning of warhead reactivity to optimize both specificity and efficiency, as demonstrated with JQ1-based BRD4-targeting cooperative covalent glues.^64^ However, simply attaching a covalent warhead does not guarantee cooperative glue function. Minimal covalent appendages on POI ligands have yielded noncooperative covalent glues with broad reactivity and off-target cellular toxicity, underscoring the importance of careful design and validation when introducing covalency into glue scaffolds.^65,66^

Covalent effector ligands can exhibit distinct pharmacological features compared to reversible ligands. Notably, their prolonged residence time can support multiple catalytic degradation cycles, with POI degradation sustained even after compound washout (Figure 4A,B). Furthermore, degradation can be driven by very low levels of effector modification, enabling the use of less optimized low-stoichiometry covalent ligands and preserving the majority of effector unmodified to support native function. For example, the covalent PROTAC BRD-SF2 (Table 2) containing a covalent VHL ligand targeting Ser110 induces prolonged BRD4 degradation in cells with minimal optimization and low modification stoichiometry, and contrasts with the activity of noncovalent PROTACs such as MZ1, which rapidly diminishes after washout.^58^ A hook effect may also be observed for cooperative covalent glues that engage effectors via trans-labeling at high concentrations, distinct from typical glues.^60,64^ While typically associated with bifunctional degraders where competitive binary binding limits ternary complex formation, sustained modification of an effector by a trans-labeling covalent glue can limit reversible binding to the POI, inhibiting the catalytic cycle and reducing degradation efficiency. It remains unclear whether all cooperative covalent glues inherently exhibit a hook effect. In the published examples to date, the scaffolds were derived from POI-binding ligands that likely occupy the binding site at high concentrations. This prevents productive interaction with the catalytically labeled effector generated in earlier trans-labeling events, leading to the hook effect from orthosteric competition. Further studies are required to determine whether this behavior is a general feature of cooperative covalent glues, and to define the threshold of binary affinity which results in a hook effect.

Covalency also provides an important practical advantage in phenotypic screening; once the effector is covalently modified in cells it can be readily identified by co-IP proteomics, facilitating rapid effector deconvolution. This contrasts with reversible glues which often require indirect methods to deconvolute MoA, such as CRISPR-based E3 ligase loss-of-function screens.^12^ This strategy has been demonstrated with electrophilic BRD9 ligands, which successfully coprecipitated DCAF16 and its binding partner DDB1.^60^

#### 2.3.2 POI-Reactive Covalent Glues

Covalent modification can also be directed to the POI, although this requires high levels of modification to be effective since such compounds lack the substoichiometric, event-driven MoA typical of effector covalent glues (Figure 4C). One example is a covalent macrocyclic glue, RMC-4998 (Table 2), which irreversibly modifies the mutant cysteine on the active GTP-bound form of KRAS^G12C^. This forms a tight inhibitory complex with cyclophilin A, demonstrating subnanomolar IC_50_ in cells and suppressing oncogenic activity in vivo (Figure 4E).^67^ Subsequent optimization yielded RMC-6921, a clinical candidate in solid tumors with superior pharmacokinetic parameters. Preclinical evidence indicates that inhibition of Ras in the ternary complex is less susceptible to resistance drivers as compared to direct inhibitors of Ras alone.^68^ To date, no examples of cooperative covalent glues modifying the POI have been reported, although these could be developed by appending covalent warheads to E3 ligands.

**Figure 5:**
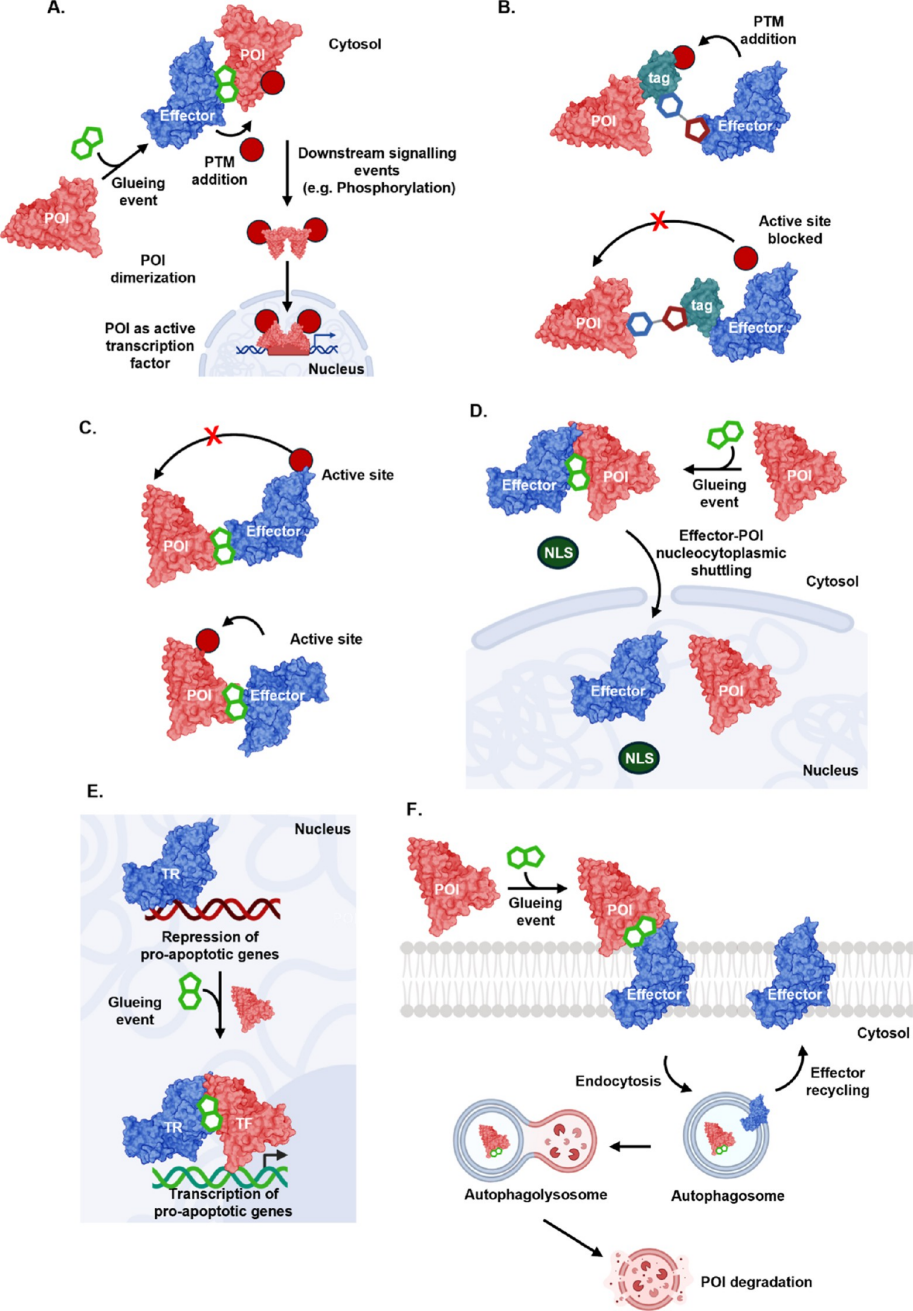
Proximity-induced pharmacology mechanisms. (A) Modulation of post-translational modifications (PTMs): addition or removal of a PTM such as phosphorylation transforms POI function to alter signaling or membrane localization. (B,C) Current challenges for generalizable PIP. (B) The majority of PIP modalities are exemplified using protein domain fusions, which may fail to generate functional ternary complexes due to tag modification or effector active site blockage. (C) The geometry of MG-induced ternary complex is critical for the recruited effector, particularly where PTMs require precise positioning of effector and POI. (D) Nucleocytoplasmic shuttling: a POI is redirected to the nucleus by proximity to an effector containing a strong nuclear localization signal (NLS), enabling nuclear import. (E) Functional relocalization to a macromolecular complex: a POI is brought into proximity with a functional binding partner, for example a transcription factor recruited to target genes to activate transcription. (F) Rewiring trafficking to degradation pathways: an endolysosomal receptor recruits a POI to a cellular pathway, leading to POI degradation in lysosomes.

It is also possible to imagine co-opting PTM pathways to install small molecule ligands covalently and site-specifically on POIs or effectors, although this concept is yet to be reduced to practice. Finally, small molecules which contain nucleophilic groups can themselves serve as substrates for ubiquitination.^69^ This mechanism expands the scope of potential POIs by enabling indirect ubiquitination of a POI ligand and offers new opportunities to modulate the proteasomal degradation pathway outside of ternary complex formation.

## 3 Unlocking Diverse Biological Outcomes through Proximity-Induced Pharmacology

While MGs aim to drug otherwise intractable targets through neomorphic PPIs, the broader concept of PIP promises a paradigm shift in drug discovery. Driven by the action of small molecules (bifunctionals or glues) generically termed “chemical inducers of proximity” (CIPs), PIP rewires interactions to create new complexes which leverage diverse classes of effectors to impart novel functions or even create new biological pathways. The feasibility of most PIP mechanisms has been demonstrated through bifunctional molecules; however, these are expected to be achievable with MGs as well, broadening the scope of induced-proximity strategies. These studies have also revealed functional, ligandable sites that can be exploited by glues. Additionally, ligands characterized during bifunctional development provide starting points for biased library design (Section 2), facilitating the discovery of glues that engage these validated sites. Here, we highlight current challenges and underexplored areas (Figure 5 and Table 3).

**Table 3:** PTM Editing and Pathway Rewiring CIPs^a^

1. CIPs mediate precise PTM editing					
PTM	modality	effector	stage	example	refs
ubiquitination	PROTAC/MGD	UPS component	clinical trial	thalidomide/ARV-471	^5,6,9^
deubiquitination	DUBTAC	deubiquitinases	SM	NJH-2–057	^85^
phosphorylation	PHICS	kinase	SM	PHICS4	^73^
dephosphorylation	PHOSTAC	phosphatase	SM	DDO3711	^80^
acetylation	AceTAG	lysine acetyltransferase	PoC	tagging	^71,72^
*O*-GlcNAcylation	OGTAC	*O*-GlcNAc transferase	PoC	tagging	^75^
arginine methylation	MrTAC	methyltransferases	PoC	tagging	^74^
*S*-deacylation		thioesterase	PoC	tagging	^82^

2. CIPs rewire cellular pathways					
pathway/location	modality	effector	stage	example	refs
lysosome	LYTAC	lysosome-targeting receptors	SM	CI-M6PR	^100^
autophagy	AUTOTAC	autophagy-lysosome system	SM	PHTPP-1304	^101^
autophagy	ATTEC	autophagosome protein	SM	LD-ATTEC1	^102^
nucleus/cytoplasm	TRAM	nuclear import/export shuttle	PoC	tagging	^96^
RNA	RIBOTAC	ribonuclease	SM	JUN-RIBOTAC	^106^
DNA	TCIP	DNA-binding proteins	SM	CDK-TCIPs	^98,99^
target cell	RIPTAC	Oncoproteins	clinical trial	HLD-0915	^105^

a SM: small molecule; PoC: proof of concept using protein tagging method.

### 3.1 CIPs Mediate Precise PTM Editing

Targeting PTM pathways has long been a focus of drug discovery, exemplified by the success of kinase inhibitors, which account for over 30% of approved targeted cancer therapies.^70^ In contrast to traditional approaches that inhibit or activate enzymes directly, PIP offers a more versatile and selective strategy by recruiting effectors to add or remove PTMs on a POI. CIPs have successfully modulated diverse PTM mechanisms (Table 3), including writers (e.g., kinases, acetyltransferases)^71–75^ and erasers (e.g., phosphatases, deubiquitinases).^76–86^ The most established CIPs are targeted protein degraders (MGs, PROTACs) which exploit ubiquitination-induced degradation,^5,6,9^ while DUBTACs recruit deubiquitinases (DUBs) to stabilize a POI.^85^ Other systems extend this concept: PHICS promote phosphorylation by engaging kinases (Figure 5A),^73^ whereas PhosTACs induce dephosphorylation via phosphatases.^76,77,80,86^ Related modalities such as AceTACs^71,72^ and OGTACs^75,87^ enable the installation of acetylation and *O*-GlcNAcylation, respectively. The feasibility of editing protein lipidation was recently demonstrated through the recruitment of protein thioesterases to *S*-acylated proteins thereby controlling membrane association, although this has yet to be demonstrated using a CIP.^82^

CIP-induced PTM changes mimic native regulatory processes, modulating protein stability, localization, or activity. In some cases, they may restore dysregulated PTM states in disease. For example, a frameshift mutation on cystic fibrosis transmembrane conductance regulator (CFTR) causes polyubiquitination and proteolysis; DUBTACs can induce CTFR deubiquitination and stabilization, acting as a potential human cystic fibrosis treatment.^85^ CIPs may in future be used to install PTMs at novel sites or on previously unmodified neosubstrates, rewiring cellular signaling networks and unlocking new biological outcomes. While engineered PTM rewiring may create novel signaling circuits capable of counteracting disease processes, the complexity of PTM networks and their context-dependent effects present a challenge for rational design of neo-PTMs.

Despite its potential, the practical application of CIPs for PTM editing faces significant hurdles due to the limited availability of suitable effector ligands and the regulation of substrate recognition. Effective CIPs target noncatalytic substrate recognition sites to enable selective effector recruitment without disrupting enzymatic function, as exemplified by E3 ligase ligands used in degraders.^9^ However, most known effector ligands are active-site inhibitors which are generally unsuitable for efficient PTM editing.^88,89^ As a result, proof-of-concept studies largely rely on engineered protein systems such as dTAG and HaloTag, where the effector and/or target are fused to proteins which can be recruited by tag-specific small molecule ligands.^90,91^ For example, the first PhosTACs used an engineered system of FKBP12^F36V^-tagged phosphatase PP2A and a Halo-fused substrate (e.g., PDCD4, FOXO3a) to induce dephosphorylation.^80^

However, these large protein tags (>20 kDa) are generally restricted to fusion at protein termini and can disrupt native structure or localization, limiting physiological relevance (Figure 5B). The recruited effector must also be correctly positioned within the ternary complex to modify the intended residue on the POI, which may not be possible with domain fusion approaches. Moreover, the biological outcome of a PTM often depends on its architecture, such as linkage type or topological arrangement (Figure 5C).^92^ For instance, K48- versus K63-linked ubiquitin chains can direct the substrate to proteasomal degradation or nondegradative signaling pathways,^93^ respectively, and achieving such precision may require multiple effectors to install the correct combination or sequence of PTMs. Similarly, the functional activation of some proteins, such as MAP kinases, requires multiple phosphorylation events that may need to be installed via processive or distributive mechanisms.^94^ In this context, functional genomics (Section 4) and machine learning approaches (Section 5) will be essential tools to identify optimal effectors and recruitment strategies.^95^

### 3.2 CIPs Rewire Cellular Pathways

Protein function is often tightly linked to its subcellular localization, and protein mislocalization can cause various pathological conditions.^95^ CIPs could correct disease-associated mislocalization,^96,97^ deliver an effector directly to a macromolecular target (e.g., a transcription factor to a target DNA region)^98,99^ or recruit a protein into specific cellular pathways by engaging subcellular localization-defining proteins.^100–104^ A recent example are Targeted Relocalization-Activating Molecules (TRAMs), which redirect protein targets into or out of the nucleus by recruiting components of the nucleocytoplasmic transport machinery (Figure 5D).^96^ While CIP-mediated relocalization can confer gain-of-function effects by recruiting proteins to compartments where they are not normally active, they may also sequester hyperactive or pathogenic proteins away from their site of action, thereby suppressing their activity. Recent examples include the cyclophilin recruiters of KRAS oncoproteins,^67,68^ and regulated induced proximity targeting chimeras (RIPTACs),^105^ which capture and restrict the function of essential proteins in the presence of overexpressed oncoproteins. However, our limited understanding of intracellular trafficking dynamics remains a key challenge in the development of relocalization-inducing CIPs, making it difficult to predict the direction of trafficking and choice of suitable effector proteins. One potential strategy is to exploit well-characterized compartment-specific scaffolding or transport proteins, such as those employed in TRAMs, where the directionality and mechanism of cargo movement are clearly defined.

CIPs have also been shown to rewire macromolecular complexes involved in the regulation of transcription. For example, bifunctional CDK–transcriptional/epigenetic chemical inducers of proximity (CDK-TCIPs) relocalize cyclin-dependent kinases (CDKs) to genomic loci repressed by the transcriptional repressor BCL6.^99^ In certain cancers, BCL6 epigenetically silences genes involved in apoptosis and cell cycle arrest (Figure 5E).^98,99^ CDK-TCIPs reverse this repression by reactivating pro-apoptotic gene expression, highlighting the therapeutic potential of site-specific enzymatic relocalization. Ribonuclease-targeting chimeras (RIBOTACs) recruit RNase L to specific mRNAs or microRNAs, enabling selective RNA degradation and transcript-specific modulation of gene expression.^106^ Induced proximity strategies can also extend beyond nucleic acids and proteins. For instance, bringing shelterin complex components to selected telomeres may enhance telomere maintenance and genome stability, with potential applications in aging and age-related diseases.^107^ The spatial organization of lipids within membranes creates unique biochemical microenvironments, presenting an opportunity to use CIPs to localize lipid-modifying enzymes to remodel specific membrane compartments, enabling targeted control over membrane composition, dynamics, and associated signaling pathways.

Redirecting a POI to specific cellular machineries can offer more controllable outcomes than general relocalization to subcellular compartments. This strategy has been demonstrated in TPD approaches that exploit the cell’s endogenous endosomal and autophagolysosomal pathways to degrade secreted, membrane-bound, cytosolic proteins, and even cytosol-resident organelles.^100,102,104,108,109^ Lysosome-targeting chimeras (LYTACs) co-opt cell–surface lysosome-targeting receptors (LTRs), such as cation-independent mannose-6-phosphate receptor (CI-M6PR), to internalize extracellular or membrane-associated POIs and direct them toward lysosomal degradation (Figure 5F).^100^ Similarly, AUTOTACs have been proposed to induce degradation by recruiting the autophagy receptor p62 to POIs, promoting POI sequestration into autophagosomes, although this has yet to be convincingly reduced to practice due to challenges in generating selective p62 ligands.^101^ Autophagosome tethering compounds (ATTECs) link POIs to LC3, a key autophagosome component, directing proteins to the autophagy-lysosome pathway for clearance.^102^ However, the development of suitable LC3 ligands for ATTECs has been proved challenging.^110^ CIPs may in future rewire secretory pathways for therapeutic benefit; for example, redirection of proteins to the exosomal secretion pathway may enable selective release of tumor-associated antigens to stimulate antitumor immune responses. While these strategies hold great promise, identifying suitable effectors and corresponding ligands or glues remains a significant challenge, and co-opting key components of essential cellular pathways may risk perturbing their native physiological roles, potentially resulting in off-target effects or toxicity.

## 4 Functional Genomics and Protein Editing to Enable MG and CIP Discovery

Chemical approaches have significantly advanced MG discovery. However, a major bottleneck remains the limited number of well-characterized, gluable effectors and compatible POIs. Emerging evidence suggests that MGs can mimic naturally occurring genetic perturbations, such as indels,^111^ missense mutations,^112^ or chemical modifications such as PTMs^113,114^ to promote or stabilize novel PPIs (Figure 6A). For example, IMiDs, the paradigm class of MG degraders, mimic a widespread C-terminal degron PTM to enable recruitment to the cognate E3 ligase, CRBN.^113,114^ Similarly, protein semisynthesis of C-terminal amide-bearing proteins (CTAPs), a naturally occurring PTM during oxidative stress, revealed the presence of a potentially gluable C-terminal amide degron binding site in the E3 ligase FBXO31.^115^ These and similar observations discussed below highlight the potential of introducing defined perturbations into effectors or POIs to uncover key structural motifs or interfaces that can be recapitulated by MGs. Such strategies could guide the systematic rational design and discovery of next-generation glues with enhanced selectivity and a range of functional outcomes.

**Figure 6:**
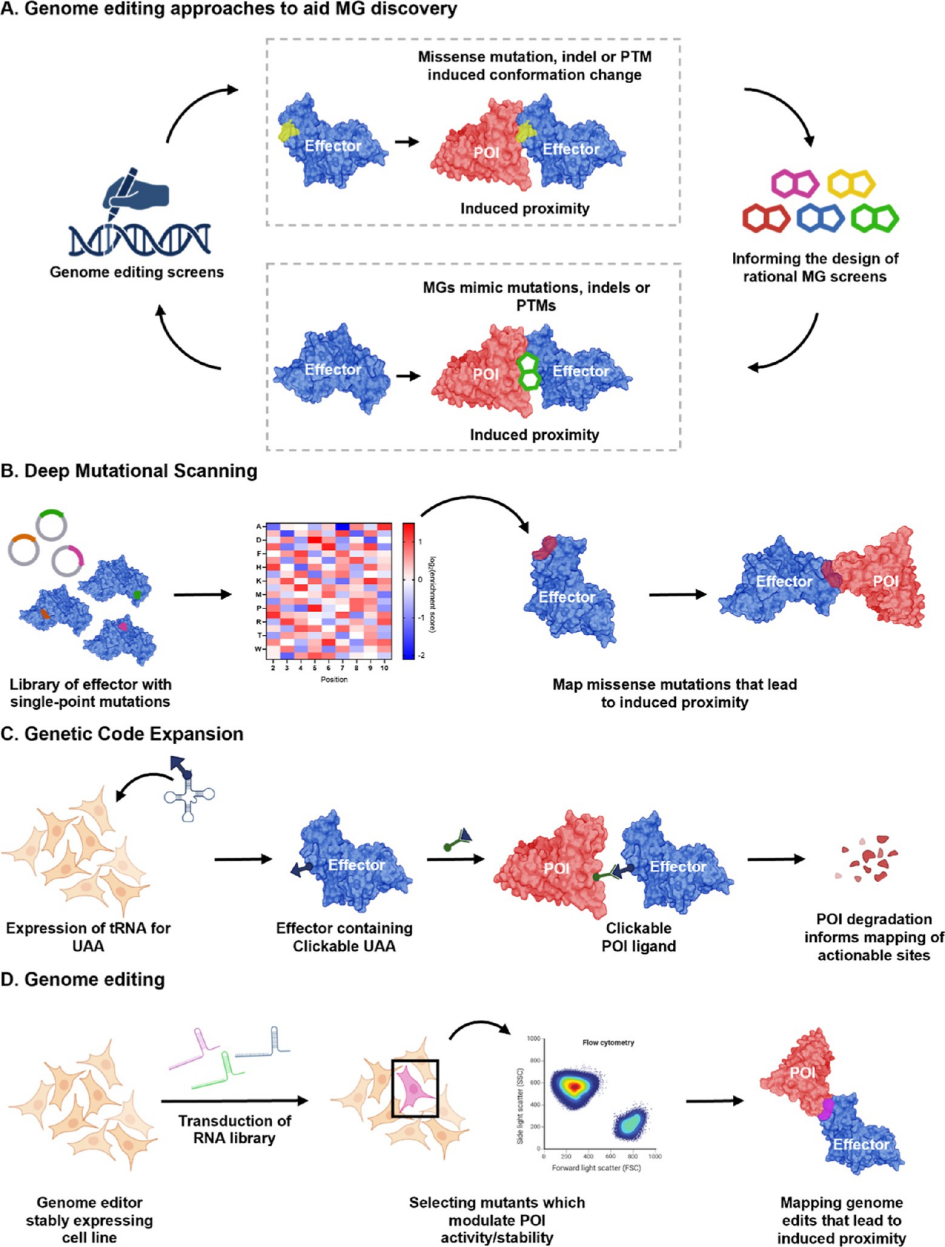
Synthetic biology and genomic technologies for MG discovery. (A) MGs promote conformational changes that facilitate ternary complex formation, often mimicking the effects of indels, missense mutations, or PTMs. These structural perturbations can be systematically explored using genome engineering and synthetic biology approaches. (B) Deep mutational scanning (DMS): systematic screening of all single-point mutants in a target protein enables identification of conformational variants that favor or hinder ternary complex formation, providing insight into potential glue–responsive interfaces. (C) Genetic code expansion: incorporation of unnatural amino acids (UAAs) at defined sites enables covalent ligand attachment, facilitating mechanistic studies of MG action and ligandability mapping of cryptic binding surfaces. (D) Genome-wide editing: CRISPR-based approaches allow functional interrogation of gene variants, isoforms, and mutational landscapes across the genome, revealing genetic contexts where MGs may be effective or uniquely selective.

### 4.1 Effector Prioritization by Customized Gene Synthesis and Deep Mutational Scanning

Advances in synthetic biology have enabled the scalable generation of custom gene libraries, allowing rapid synthesis of modified genes. Gain-of-function gene variants can artificially induce proximity, facilitating the study of effector mechanisms or functional outcomes on POIs. A recent study developed a pooled ORFeome library wherein open reading frames were fused to proximity-inducing domains and transiently overexpressed in cells,^116^ supporting HTS for functional effectors that induce degradation or stabilization of a reporter protein. Another approach combined CRISPR/Cas knock-in endogenous E3 ligase tagging with HaloTag domains and used bifunctional-induced proximity screening to identify candidate TPD effectors.^117^ This strategy avoids the artifacts caused by overexpression which can overwhelm the degradation machinery but is limited by varying endogenous effector expression between cell lines. As previously noted, large fusion protein tags disrupt native effector structure which may complicate interpretation and limit translational relevance, as the large tags are unlikely to mimic CIP mechanisms.

Deep mutational scanning (DMS) provides a powerful approach to systematically interrogate protein function at single-residue resolution (Figure 6B).^118,119^ Using de novo DNA synthesis, libraries encoding all possible amino acid substitutions at selected sites or across entire proteins can be constructed to study the effects of nonsynonymous variants on protein stability, activity, and interactions. A gain-of-function mutation that promotes a neomorphic PPI coupled to a functional readout for induced proximity (e.g., induced degradation of a POI) can identify gluable surfaces, and model conformational changes which could be induced by a MG at the same site.^111^ For example, DMS was used to systematically study functional TPD hotspots and resistance mechanisms to small-molecule degraders on CRBN and VHL,^120^ as well as to identify ligandable cysteines and residues governing selectivity between DCAF16 and FBXO22.^121^ Gene synthesis is not limited to point mutations, but can also be used to introduce deletions and insertions into native and designed proteins. Naturally occurring medulloblastoma-associated insertions and substitutions in the E3 ligase substrate receptor KBTBD4 were incorporated using DMS to demonstrate convergence with the MoA of MG UM171. UM171 mimicked disease-associated mutations leading to degradation of HDAC1-associated transcriptional corepressor CoREST, making a compelling case for systematic mutational analysis for discovery of neomorphic sites in MG discovery.^111,122^ This concept has recently been expanded to a generalized screening platform which systematically varies loop sequences in effector or POI across very large encoded libraries, to identify potentially gluable neo-interacting surfaces.^124^

### 4.2 Proteome-Wide, Site-Specific Proximity Induction Using Genetic Code Expansion

Genetic code expansion offers a systematic solution to identify ligandable sites through site-specific incorporation of unnatural amino acids (UAAs) bearing handles for strain-promoted bioorthogonal ligation (Figure 6C).^123^ These engineered residues can be selectively conjugated to chemically functionalized ligands in cells via click chemistry, allowing precise in situ protein functionalization. Our group harnessed this strategy to identify functionally actionable effector sites by tethering POI ligands onto E3 and E2 proteins to induce proximity-driven POI degradation in an approach termed site-specific ligand incorporation-induced proximity (SLIP).^21^ In contrast to the inflexible approach of domain fusions, SLIP allows functional mapping of effectors at single-residue resolution, revealing degradation-competent ligandable sites, such as Cys111 on SOCS2, priming the rational design of PROTACs or MGs. Compared to DMS, SLIP offers a more logical and versatile method to probe PIP; similar strategies (ELF and BPI) have been demonstrated by other groups for both effector and POI, underlining the versatility of this strategy.^124–126^

While originally developed in the context of TPD, SLIP and related platforms are applicable to functional site identification for other classes of CIPs provided a phenotypic readout is available, including the full range of PIP modalities described in Section 3. Despite its precision, a limitation of SLIP, as with DMS, is the need to individually construct each variant on a plasmid, limiting scalability. This may be addressed by ongoing advances in gene synthesis platforms, or by prioritizing candidate sites using structural databases,^127^ ligandability prediction tools,^128^ or machine learning–based models.^129^ Another challenge is the reliance on exogenous overexpression systems, which may yield artifacts due to absent or insufficient endogenous cofactors or binding partners. To mitigate this, functional validation across multiple cell lines with diverse proteomic contexts is essential. Additionally, the integration of genome editing technologies may enable site-specific manipulations at endogenous loci, preserving physiological relevance and improving translational potential.

### 4.3 Proteome-Wide Genome Editing Using CRISPR

Gene editing offers high programmability, typically requiring only short oligonucleotides to guide enzymes to specific genomic loci.^130^ This enables scalable library generation targeting thousands of sites combined with next-generation sequencing (NGS) as a high-throughput readout (Figure 6D). For example, CRISPR-Cas9 knockout (KO) screens targeting all nonessential components of the ubiquitin-proteasome system (UPS), combined with phenotypic selection tools such as FACS, have uncovered effectors in MG degrader mechanisms.^12,131^ However, such screens require clear, quantifiable phenotypes; while this is easily achieved for TPD or stabilization by monitoring protein expression, other PIP modalities may require more sophisticated or context-dependent readouts.

Recent innovations, including CRISPR Oligo Recombineering (CORe),^132^ prime editing (PE),^133^ and base editing (BE),^134^ move beyond simple KO screens to edit protein primary sequences. The CORe platform applies single-stranded DNA templates to precisely map sites with minimal introduction of random insertions and deletions (indels).^132^ PE uses a Cas9 nickase and reverse transcriptase to introduce diverse edits without relying on indel-prone homology-directed repair (HDR), supporting systematic substitutions and insertions at any amino acid.^135^ PE precisely inserted a zinc finger degron sequence in-frame of POI-coding genes in human cells to enable MG-induced POI degradation.^136^ Such high-fidelity editing is currently limited by low efficiency to haploid models, although this may not be a significant barrier for neomorphic screens since a single edit in a diploid system may be sufficient to drive measurable gain-of-function in an effector-POI interaction. In contrast, BE fuses catalytically inactive Cas9 to a base editing enzyme to allow targeted single-nucleotide changes directly on the genome without the risk of indels, and access to certain amino acid substitutions at scale. For example, BE was used to explore the essentiality of over 13,800 ligandable cysteines across 1750 cancer genes.^137^ BE scanning, in parallel with DMS approaches, also revealed the HDAC1–KBTBD4 interface stabilized by the glue UM171, as discussed in Section 4.1.^111,122^ However, current limitations in BE design and precision restrict possible edits to C → T or A → G, with potential for bystander edits at several bases either side of the target site.^138^ Combining BE and PE has the potential to expand mutational scope and precision,^139^ and together with CORe hold huge potential for genome-wide discovery of cryptic functional sites for future MG development.

## 5 AI/ML-Approaches May Enable de Novo Glue Design

Recent advances in AI and machine learning (AI/ML) are rapidly transforming drug discovery, with growing potential to support rational MG design. While there are increasing examples of successful traditional ligand discovery for single targets, MGs pose unique challenges due to the vast search space, encompassing chemical space, protein complex geometry, and all possible neomorphic interactions within the ternary complex. Combined ligand- and protein-centric approaches are increasingly being applied to this problem, although few models to date have been tailored to MGs (Figure 7A).

**Figure 7:**
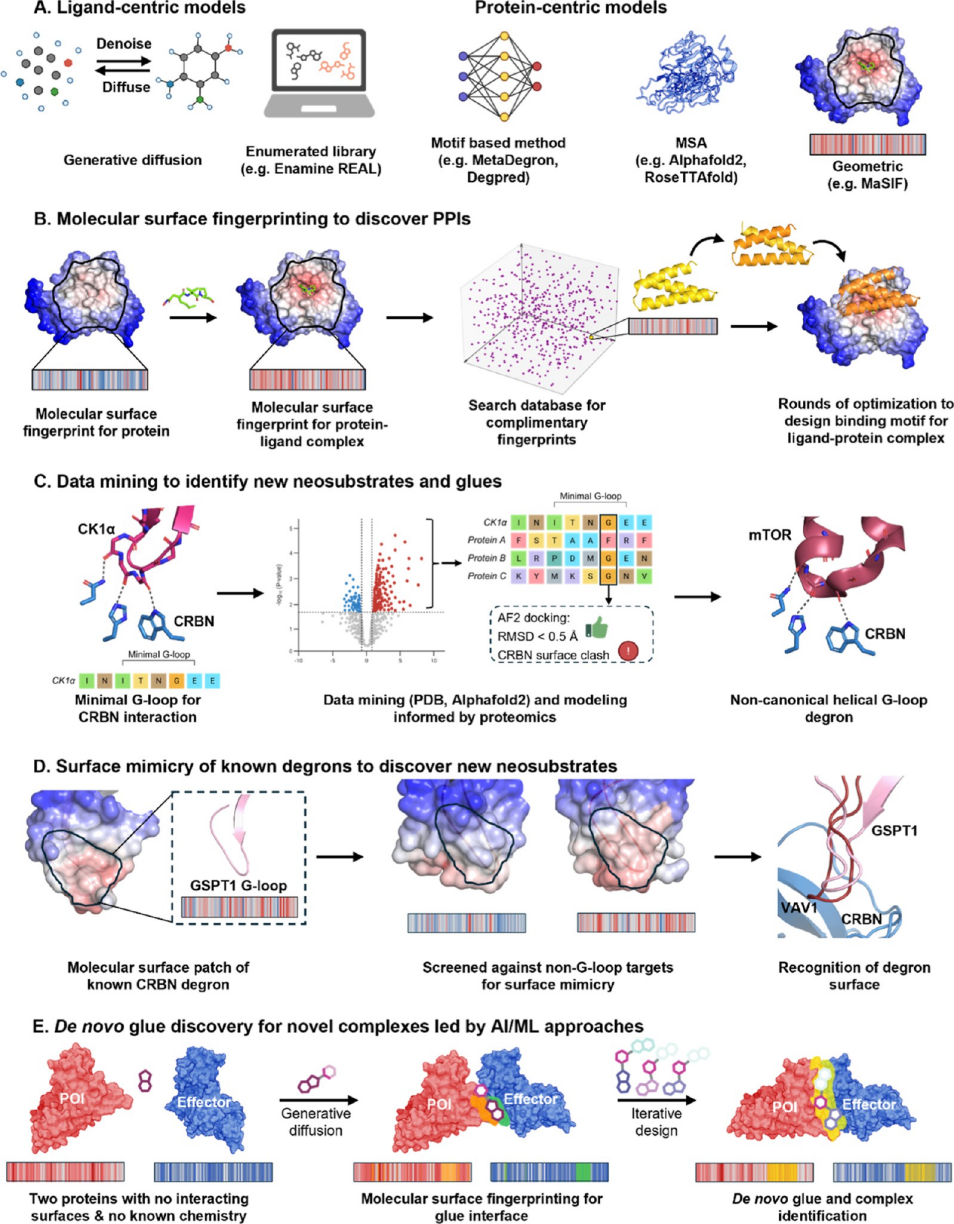
AI/machine learning for molecular glue discovery. (A) Ligand-centric models focus on the chemical and structural properties of candidate compounds to predict glue-like activity. Protein-centric models analyze structural features, interaction surfaces, and dynamics of target proteins to identify ligandable or neomorphic glue–responsive interfaces. Together, these methods support the prioritization of compound–protein pairs and guide rational MG design. (B) Molecular surface fingerprinting (MaSIF) predicts interactions based on surface and physicochemical properties. MaSIF-neosurf designed a protein helix to bind to a protein–ligand complex. (C) Computational data mining of Alphafold and cross-referencing to proximity-based proteomics expanded the canonical β-helical G-loop recognition motif of CRBN to a minimal helix. (D) MaSIF analysis of the β-helical G-loop degron against therapeutic targets of interest identified that CRBN can recognize a degron surface, rather than a specific secondary sequence. (E) In future, de novo glue design may be achieved by combining geometric protein modeling software such as MaSIF with generative diffusion models for ligand design. Ligand-centric AI/ML methods from conventional drug discovery are applicable to glue screening (Section 2) and facilitate access to expanded chemical space. Virtual screening of massive enumerated libraries (e.g., Enamine REAL, 10 billion compounds conforming to drug like space) can identify ligands for biased or D2B libraries,^140^ while QSAR models can accelerate hit optimization and selectivity profiling across neosubstrates,^141^ and DEL-ML models can prioritize hits and simplify deconvolution by stepping directly into commercially available compound space.^142,143^ Generative diffusion models can assist in vector design for DELs and elaboration of covalent ligands from fragment screens.^144^ Encoded library screening can rapidly generate large data sets which support iterative model refinement and transfer learning to other targets, particularly if the libraries contain privileged scaffolds.

Protein-centric methods are most useful for a preexisting effector-POI of known affinity. Degron motif predictors such as MetaDegron^145^ or Degpred^146^ can guide effector-POI selection for biochemical screens. Integration with PTM and disease mutation databases, and functional genomic data (Section 4) may further train models to identify tractable neomorphic surfaces. Tools such as AlphaFold2 ^127^ and RoseTTAFold^147^ which infer 3D protein structure from multiple sequence alignments (MSA) have the potential to predict neomorphic interfaces, although current models struggle to predict de novo protein interactions or small molecule-induced structural changes, presumably because such non-native interactions are poorly represented in native sequences. Benchmarks suggest that AlphaFold2 and AlphaFold3 perform poorly on ternary complexes formed by PROTACs.^148,149^ Geometric deep-learning platforms such as molecular surface interaction fingerprinting (MaSIF) may be more successful. MaSIF predicts interactions based on surface shape and physicochemical complementarity and has been used to design new PPIs^150^ as well as a novel protein that interacts with a ligand-induced neomorphic surface (MaSIF-neosurf) in cells (Figure 7B).^151^

The progression of the field toward an integrated AI/ML and experimental paradigm is demonstrated by a recent publication from Monte Rosa.^20^ Computational mining of protein sequences was combined with large scale affinity-based biased library screening to uncover a new minimal degron motif for CRBN (Figure 7C). This was further expanded using MaSIF to discover structurally distinct recognition motifs with molecular surfaces that emulate the canonical G-loop degron (Figure 7D). This highlights the importance of integrating multiple protein-centric approaches—including known neomorphic surfaces—for successful MG discovery. While this was demonstrated for CRBN, which benefits from well-characterized MGs and protein interfaces, a similar strategy may be generalizable. Combining protein-centric interaction predictors like MaSIF with ligand-centric generative diffusion models which design small molecules for these protein surfaces, may offer a powerful approach for future de novo MG design for a novel complex (Figure 7E).^152,153^

Despite the promise of AI/ML, model accuracy depends heavily on training data. Most models fail to predict activity cliffs, which are likely exacerbated in glue design.^154^ Protein structure training sets rely on PDB data, where glue-mediated ternary complexes are scarce. This limits predictive power, especially for dynamic or “undruggable” proteins, which are the highest value targets for CIP and MGs. Furthermore, as previously noted, ternary complex formation does not always equate to functional outcomes, and while AI/ML may predict complex formation, biological effects are harder to anticipate; integrating gene editing data (Section 4) to identify functional gluable sites at the outset may help bridge this gap. Ultimately, success will depend on combining AI/ML with large-scale genetic and omics data, high-throughput structure determination, rational screening platforms, and physically accurate modeling to incorporate protein flexibility. Iterative cycles of experimental validation and model refinement will be key to advancing de novo MG discovery, supported by new initiatives such as OpenBind in the UK.

## 6 Outlook

Molecular glue discovery is currently transitioning from a largely serendipitous endeavor to a rational design-driven discipline, through an intermediate exploration of innovative approaches. For much of the first three decades, progress was defined by a small number of landmark examples, with new glues emerging through extensive empirical screening or retrospective mechanistic insight. However, recent advances in chemical biology, functional genomics, and computational modeling are now converging to support systematic strategies for de novo discovery. Much activity to date has been obscured by the commercial excitement around MGs as potential therapeutics, leading to large investments in multiple startup companies which have been understandably slow to publish their progress. However, in contrast to the dozens of PROTACs already in clinical trials,^155^ the number of companies taking novel glues into the clinic remains small. Key challenges clearly remain, particularly around optimal screening approaches, the role of pre-existing affinity, and the prospect of true de novo structure-guided MG design.

A central question is whether some degree of intrinsic affinity between the effector and the protein of interest is required for discovery of novel MG activity. Many known glues appear to stabilize latent or weak interactions that already exist in the proteome, and unbiased screens suffer from low or zero hit rates. For many PIP modalities, such as the recruitment of enzymes to install PTMs, precise spatial orientation in the ternary complex may be essential, making discovery of functional MGs still more challenging. In this context, functional genomic approaches such as SLIP^21^ provide valuable stepping stones to identify tractable starting points. In contrast, glues that stabilize nonproductive or abortive interactions may act independently of preexisting contacts, such as covalent MGs targeting KRAS.^67^

Multiple strategies are beginning to address chemical space constraints. Structure-guided approaches have shown promise where interaction surfaces and exit vectors are well characterized, as in the cases of CRBN,^20^ FKBP12,^42,156^ and 14-3-3.^25,26^ Encoded library technologies can enable direct selection for ternary complex formation, with an emphasis on binding cooperativity and kinetic properties such as low dissociation rate, which are increasingly recognized as key predictors of functional activity.^42,43^ The integration of covalency, particularly when proximity-induced, expands the scope by enforcing selective and durable engagement, even in the absence of high intrinsic affinity.^60,61^ As the field broadens, robust validation of new mechanisms is essential. Widely used model systems such as BRD4 or FKBP12 provide useful benchmarks but may not represent the challenges found in less tractable targets, and their behavior in degradation studies may limit their utility as a generalizable standard in TPD. For example, FKBP12 may be stabilized by dTag ligands,^157^ while BRD4 appears to be prone to facile degradation.^47^

The best screening strategies remain a topic of active debate. Phenotypic screening offers the advantage of detecting functional outcomes without prior assumptions and is easily implemented for TPD provided the appropriate controls are adopted,^30^ but often leads to the repeated identification of a narrow subset of E3 ligases.^19^ This may reflect catalytic efficiency rather than genuine tractability and highlights the need to diversify effector space through POI-focused or rationally guided approaches. Direct-to-biology synthesis,^37^ functional genomic screening, and site-specific perturbation methods such as deep mutational scanning or base editing^111,122^ are beginning to enable these necessary paradigm shifts. Meanwhile, machine learning and molecular modeling are starting to predict neomorphic interfaces to prioritize compound screens and propose new MG design rules based on growing experimental data sets.^127,151,158^ CRBN once again provides the best example to date for in silico glue discovery, thanks to a large body of existing data which enables targeted screening against neosubstrates with the potential to mimic a consensus neomorphic surface.^20^ However, this work was critically enabled by a vast proprietary database and compound library, and it remains to be seen whether this can be replicated for other effectors.

Looking to the future, the broader field of proximity-induced pharmacology is emerging as a versatile therapeutic platform. Modalities such as RIPTACs,^105^ ADC-glues,^159^ and BACTACs^160^ illustrate how small molecules can redirect proteins to modulate trafficking, localization, or enzymatic activity. Some of these approaches are in clinical trials, while others are being applied to programmable systems such as CAR-T cells^161^ or CRISPR-based tools.^162^

Together, these developments point to a future in which molecular glues can be discovered more predictably and applied more broadly. Achieving this goal will require continued progress in screening technologies, deeper exploration of effector and substrate landscapes, and tighter integration of chemical, biological, and computational methods. As these approaches mature, molecular glues have the potential to become a foundational modality within proximity-based therapeutic design and offer a versatile tool to explore biology.
